# Analysis of in vitro chemoresponse assays in endometrioid endometrial adenocarcinoma: an observational ancillary analysis

**DOI:** 10.1186/s40661-016-0032-7

**Published:** 2016-12-01

**Authors:** Brittany A. Davidson, Jonathan Foote, Stacey L. Brower, Chunqiao Tian, Laura J. Havrilesky, Angeles Alvarez Secord

**Affiliations:** 1 Division of Gynecologic Oncology, Duke University Medical Center, Duke University, DUMC Box 3079, Durham, NC 27710 USA; 2 Product Development, Helomics Corporation, Pittsburgh, PA USA

**Keywords:** Endometrial cancer, Chemosensitivity, Tumor grade, Endometrioid

## Abstract

**Background:**

Chemotherapy plays a role in the treatment of endometrioid endometrial cancer (EEC); however, tumor grade may affect response. Our objective was to evaluate associations between tumor grade and in vitro chemoresponse.

**Methods:**

We conducted an analysis of primary tumor samples from women with EEC undergoing in vitro chemoresponse testing. Results were classified as sensitive (S), intermediate (I), or resistant (R) to each drug tested. Correlations between tumor grade and response were examined.

**Results:**

Data was collected from 159 patients: 28 with grade 1 (18%), 52 with grade 2 (32%), and 79 (50%) with grade 3 tumors. Median age of patients was 62 (range 31–92). Most patients were Caucasian (83%) with advanced disease (Stage III: 50.9%; Stage IV: 13.2%). Overall chemoresponse was similar across all grades. Fifty percent, 56 and 51% for grade 1, 2, and 3 tumors, respectively, demonstrated S results to at least 1 agent. There was no association between grade and in vitro response to chemotherapy agents (*p* > 0.05) except a marginal association between grade and doxorubicin response (*p* = 0.08). Grade 1 and 2 cancers were more likely to demonstrate R results for doxorubicin compared to grade 3 cancers (G1: 19% vs G2: 25% vs G3: 8%; *p* = 0.08). In a subset tested for all 7 agents, only one patient tumor was pan-R and 4 were pan-S.

**Conclusions:**

Based on our data, grades 1–3 EEC have similar in vitro chemoresponse. These findings suggest that chemotherapy may be useful in advanced low grade EECs, but further clinical correlation is needed.

## Background

Endometrial cancer (EC) is the most common gynecologic malignancy, with nearly 55,000 new cases and more than 10,000 deaths predicted for 2015 [1]. While 5 year survival trends have improved for other gynecologic malignancies, the survival rate for patients diagnosed with EC between 2004 and 2010 is lower than that of patients diagnosed between 1975 and 1977 (83 vs. 87% *p* = <0.05) [1]. Undoubtedly, many factors account for this trend; however it is yet unknown if chemoresistance plays an important role.

The mechanism underlying chemoresistance in EC is uncertain, however current dogma suggests that low grade Type I endometrioid endometrial cancers (EEC) are less likely to respond to chemotherapy. Type I tumors represent the majority of sporadic EC, characterized predominantly by endometrioid histology and expression of estrogen and/or progesterone receptors [2]. In contrast, Type II EC is less common and often of serous or clear cell histology, arising in atrophic endometrium, rather than estrogen excess [3]. There is conflicting data regarding chemotherapy response and tumor grade and histology in endometrial cancers.

Response rate (RR) to chemotherapy was not significantly different between endometrioid (44%), clear cell (32%) and serous tumors (44%) in a pooled analysis of patients with advanced or recurrent EC treated on 1 of 4 GOG trials (GOG 107, GOG 139, GOG 163, GOG 177) (clear cell *p* = 0.13; serous *p* = 0.99) [4]. In a subgroup analysis of over 600 patients with endometrioid histology alone enrolled in these same 4 GOG trials, grade 3 tumors had an estimated odds of response of approximately 1.5 times that of grade 1 tumors, although these results were not statistically significant (*p* = 0.09) [4]. However, these two analyses were based on a retrospective assessment of investigator-determined response which may be prone to subjective assessment and error.

We previously explored the association between tumor grade and cytotoxic treatment response in patients with advanced or recurrent EEC (*N* = 91). Contrary to expectations, grade 2 cancers were more likely to respond to all types of chemotherapy (72 vs 43% *p* = 0.02) and to carboplatin/paclitaxel doublets (72 vs 41% *p* = 0.02) compared to grade 3 cancers [5]. However, this study was limited by lack of central pathology review, small sample size, and paucity of patients with grade 1 ECC.

Given the contradictory data and limitations of prior studies, we explored in vitro chemoresponse profiles to obtain insight into the relationship between chemotherapeutic anti-tumor activity and grade in EEC specimens from women enrolled in observational studies.

## Methods

The study population included women with endometrioid endometrial cancer whose primary cancer specimens were submitted for in vitro chemoresponse assay testing on prospectively-accrued observational studies between 2006 and 2010. These were longitudinal, observational multi-center studies examining the outcomes associated with chemosensitivity assays in women with gynecologic malignancies. Participants had not received chemotherapy prior to specimen collection. Tumor grades were assigned by the institutions submitting the specimens for testing. Assays were conducted for up to 7 cytotoxic agents including carboplatin, cisplatin, doxorubicin, paclitaxel, docetaxel, gemcitabine, and topotecan.

Details regarding the particular chemoresponse assay used in this study (ChemoFx®, Helomics Corporation, Pittsburgh, PA) have been described elsewhere [6, 7]. Assay preparation included an immunocytochemistry step to aid in the confirmation that cells were of epithelial, rather than stromal, origin. All cultures required a majority of epithelial cells to proceed to chemoresponse testing. A board-certified pathologist assessed cell morphology.

Inhibition of tumor growth was measured at several serially-diluted concentrations of each cytotoxic agent tested. For each drug, the area under the dose–response curve (AUC) was calculated. Greater sensitivity to the therapy tested was indicated by a smaller AUC. Using established criteria, tumor chemoresponse was classified using the in vitro AUC score into one of three categories: sensitive (S), intermediate sensitive (I), or resistant (R). The in vitro tumor response rate (RR) for each agent was then defined as the proportion of patients with tumors testing either S or I for that agent.

The primary endpoint of this ancillary study was to assess the association between tumor grade and in vitro chemoresponse assay results in EEC. Patient demographics were also collected, including age and stage at diagnosis. Correlations of tumor grade with assay results were examined using Cochran-Armitage test for trend using SAS version 9.4 (SAS Institute, Cary, NC).

## Results

A total of 159 patients were included for this analysis. Twenty-eight patients had grade 1 (18%), 52 had grade 2 (32%), and 79 (50%) had grade 3 tumors. The median age of patients was 62 (range 31–92). Most patients were Caucasian (83%) and had advanced stage disease (Stage III: 50.9%; Stage IV: 13.2%) at diagnosis [Table 1].

**Table 1:** Patient Characteristics by Tumor Grade

	Grade 1 (*n* = 28)	Grade 2 (*n* = 52)	Grade 3 (*n* = 79)	Total (*n* = 159)
	No. (%)	No. (%)	No. (%)	No. (%)
Age (years)				
Median (Range)	63.5 (42–92)	62 (32–87)	62 (31–89)	62 (31–92)
< 50	2 (7.1)	8 (15.4)	9 (11.4)	19 (11.9)
50–64	13 (46.4)	22 (42.3)	36 (45.6)	71 (44.7)
65–74	8 (28.6)	14 (26.9)	24 (30.4)	46 (28.9)
≥ 75	5 (17.9)	8 (15.4)	10 (12.7)	23 (14.5)
Race				
White	24 (85.7)	45 (86.5)	63 (79.7)	132 (83)
Black	1 (3.6)	4 (7.7)	12 (15.2)	17 (10.7)
Other	3 (10.7)	3 (5.8)	4 (5.1)	10 (6.3)
FIGO Stage				
I	11 (39.3)	8 (15.4)	21 (26.6)	40 (25.2)
II	0 (0.0)	8 (15.4)	7 (8.9)	15 (9.4)
III	15 (53.6)	28 (53.8)	38 (48.1)	81 (50.9)
IV	2 (7.1)	6 (11.5)	13 (16.5)	21 (13.2)
Unknown	0 (0.0)	2 (3.8)	0 (0.0)	2 (1.3)

As mentioned previously, the chemotherapeutic agents tested in this assay included carboplatin, cisplatin, doxorubicin, and paclitaxel, which are commonly used in EC. In addition, other cytotoxics incorporated in the panel were evaluated including docetaxel, gemcitabine, and topotecan. Not all patients had their tumors tested for all 7 cytotoxic agents. Assay results were available for the following cytotoxic agents stratified by grade 1, 2, and 3: carboplatin (25/28[89%], 41/52[79%], 66/79[84%]), paclitaxel (24/28[86%], 40/52[77%], 65/79[82%]), doxorubicin (27/28[96%], 48/52[92%], 77/79[97%]), cisplatin (24/28[86%], 43/52[83%], 67/79[85%]), docetaxel (19/28[68%], 35/52[67%], 61/79[77%]), gemcitabine (14/28[50%], 26/52[50%], 45/79[56%]), and topotecan (22/28[71%], 41/52[79%], 61/79[77%]) [Table 2].

**Table 2:** In Vitro Tumor Responses to Seven Drugs

Assay Result^b^						
Drug	Tumor Grade	No. patients	R (%)	I (%)	S (%)	*P* value^a^
Carboplatin						.928
	1	25	28.0	40.0	32.0	
	2	41	24.4	39.0	36.6	
	3	66	19.7	53.0	27.3	
Cisplatin						.322
	1	24	29.2	41.7	29.2	
	2	43	18.6	55.8	25.6	
	3	67	13.4	58.2	28.4	
Docetaxel						.812
	1	19	26.3	42.1	31.6	
	2	35	8.6	54.3	37.1	
	3	61	18.0	47.5	34.4	
Doxorubicin						.080
	1	27	18.5	55.6	25.9	
	2	48	25.0	56.3	18.8	
	3	77	7.8	61.0	32.2	
Gemcitabine						.238
	1	14	28.6	28.6	42.9	
	2	26	30.8	23.1	46.2	
	3	45	22.2	62.2	15.6	
Paclitaxel						.670
	1	24	33.3	33.3	33.3	
	2	40	15.0	37.5	47.5	
	3	65	15.4	53.9	30.8	
Topotecan						.861
	1	22	27.3	45.5	27.3	
	2	41	24.4	48.8	26.8	
	3	61	23.0	50.8	26.2	

^a^Correlation of tumor grade with assay result examined by Cochran-Armitage test

^b^ *R* resistant, *I* intermediately sensitive, *S* sensitive

The number of R results, defined as responses that were neither S nor I, was similar across grades. Twenty seven percent, 21, and 17% for grade 1, 2, and 3 tumors, respectively, demonstrated resistant chemoresponse results for any of the agents tested. There was no association between tumor grade and in vitro response to various chemotherapy agents (*p* > 0.05) other than a marginal association between grade and response to doxorubicin (*p* = 0.08) (Table 2). Specifically, grade 1 and 2 cancers were more likely to demonstrate R assay results for doxorubicin compared to grade 3 cancers (G1: 18% vs G2: 25% vs G3: 8%; *p* = 0.08).

**Fig. 1:**
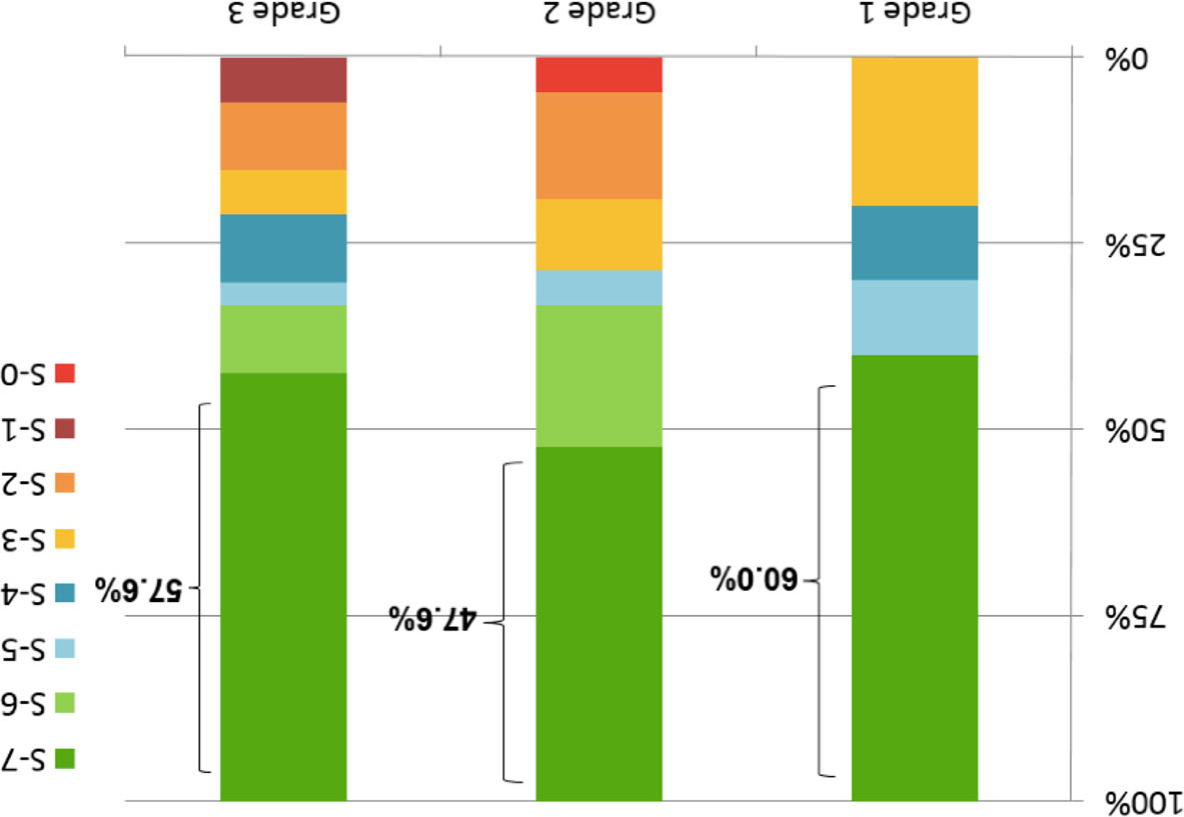
Distribution of assay results across seven single agent treatments. Patients (*n* = 64) were categorized based on response to carboplatin, cisplatin, doxorubicin, gemcitabine, paclitaxel, docetaxel, and topotecan. Tumors were either resistant to all drugs (S0), or sensitive (S + I) from anywhere from one (S1) agent to all 7 agents (S7). Sixty percent of grade 1 tumors were pan-sensitive (S7) vs 47.6 and 57.6% of grades 2 and 3

Ten of 28 (36%) grade 1, 21/52 (38%) grade 2, and 33/79 (42%) grade 3 tumors were tested for all 7 cytotoxic agents (Fig. 1). Of those grade 1 tumors tested for all 7 cytotoxic agents, 6/10 (60%) were pan-sensitive (defined as S + I), and none were pan-resistant. Of the 21 grade 2 tumors tested for all 7 agents, 10 (47.6%) were pan-sensitive and 1 (5%) pan-resistant. Similarly, 19 of 33 (57.6%) grade 3 tumors were pan-sensitive while none were pan-resistant. When examining the entire cohort of tumors, 14/ 28 (50%) grade 1 tumors exhibited a sensitive (S only) chemoresponse assay to at least 1 agent. Similarly, 29/52 (56%) of grade 2 tumors and 40/79 (51%) grade 3 tumors also showed in vitro sensitivity to at least one cytotoxic agent. If S + I responses are considered, these RRs increase to 73, 79 and 83% for grades 1, 2 and 3 tumors, respectively.

## Discussion

Our data suggests there is no difference in in vitro chemoresponse among EECs of various grades. When stratified by tumor grade, RRs were similar for 7 chemotherapeutic agents with the exception of doxorubicin, where grade 3 tumors exhibited a non-significant increased RR (92% (S + I)) compared to grade 1 (81%) and grade 2 (75%) tumors. Overall our results are similar to the clinical findings reported by McMeekin and colleagues examining the relationship between tumor histology and chemotherapeutic response in 4 GOG endometrial cancer trials (GOG#107, GOG#139, GOG#163, GOG#177). To date, this GOG ancillary analysis is the largest study to examine the association between tumor grade and chemotherapy response in EEC [4]. These trials encompassed both advanced and recurrent EC treated with a variety of cytotoxic agents, including doxorubicin, a doxorubicin/cisplatin doublet, a doxorubicin/paclitaxel doublet, and a doxorubicin/cisplatin/paclitaxel triplet administered in various intervals and doses [8–11]. The majority of patients included in these studies had also received prior radiation therapy. In a subgroup analysis of over 600 patients with endometrioid histology only, there was no difference in response of grade 3 versus grade 1 tumors (*p* = 0.09). Furthermore, tumor grade was not associated with progression free survival (PFS) or overall survival [4]. This may be due, in part, to the fact that patients had advanced or recurrent disease associated with a poor prognosis, limiting the ability to detect differences in subgroups. While there was heterogeneity among these various studies, the strength of the ancillary analysis of the GOG trials is the uniform delivery of therapy in a prospective clinical trial setting, and pathologic confirmation of tumor grade and histology required for enrollment.

The correlation between in vitro chemoresponse & tumor grade, as well as the correct definition of this response (S + I vs S) in women with EC is unknown. We compared our in vitro chemotherapy response to the RR reported in the literature for combination carboplatin/paclitaxel (CT) or cisplatin/doxorubicin/paclitaxel (TAP), the current standard of care therapy for advanced or recurrent EC. In our study, RRs based on in vitro chemotherapy response defined as S + I, were noted for the following agents: carboplatin, 77%; paclitaxel, 81%; cisplatin, 82%; and doxorubicin, 85%. These in vitro chemotherapy RRs are nearly twice the RRs reported in the 4 GOG trials studied by McMeekin et al. [4] However, when only S results are considered (carboplatin, 31%; paclitaxel, 36%; cisplatin, 28%; doxorubicin: 26%), our in vitro results are similar to those reported in several small phase II trials evaluating the use of single agents in chemo naïve patients with EC. For example, 20% of women with chemo naïve advanced or recurrent EC receiving single agent cisplatin achieved a response [12], compared to a 28% in vitro response in our analysis. Furthermore, our in vitro results were nearly identical to those seen with paclitaxel (14.3% CR, 21.4% PR) in GOG-86O and doxorubicin (RR 25%) in GOG-107 [10, 13]. No in vitro doublet assays were performed for comparison with in vivo studies. Comparisons between the populations in these prior studies and our results are limited due to different dosing schedules as well as the in vitro vs. in vivo differences. However, the results suggest that in vitro studies may be applicable in predicting tumor response in vivo.

Data from epithelial ovarian cancer (EOC) specimens have compared in vitro assay response to clinical outcomes. Krivak and colleagues reported that in vitro assay resistance to carboplatin is associated with decreased PFS in women with advanced-stage EOC treated with platinum based therapy. Specifically, women whose tumor specimens demonstrated in vitro platinum resistance were at higher risk for disease progression compared to those with sensitive or intermediate sensitive assay results (median PFS: 11.8 vs 16.6 months, respectively, *P* < .001) [6]. In addition, our group compared in vitro assay response between Type I and Type II EOC and found that, despite the dogmatic belief that Type I EOC are chemoresistant, the majority (86%) of Type I tumors were chemosensitive to at least one cytotoxic agent and 35.7% were pan-S to all 7 agents tested [14]. Multi-drug resistance was twice as likely in women with Type I EOC compared to Type II EOC (pan-R, 14.3 vs. 6.8% (*p* = 0.268); pan-S, 35.7 vs. 51.2% (*p* = 0.183)), but did not reach statistical significance. Similarly, in our analysis in EC, 20% of grade 1 tumors were pan-sensitive. None of the grade 1 ECs were resistant to the 7 agents included in the cytotoxic panel, indicating that chemotherapy may be useful in the treatment of grade 1 advanced or recurrent disease. These recent studies demonstrate the clinical validity and utility of in vitro chemotherapy assays to direct therapy. Continued evaluation could further support the role of this test in clinical practice, especially given the interest in precision and personalized medicine.

Understanding the intricacies of molecular differences in EC histologies may be fundamental in directing targeted therapies. Data from The Cancer Genome Atlas (TCGA) have identified molecular signatures that may account for heterogeneity in treatment response for endometrioid endometrial tumors. While only a small percentage of grade 1 and 2 EECs have genetic fingerprints similar to serous endometrial cancers, almost 25% of grade 3 EECs possess signatures closely related to these more aggressive tumors [15]. This data supports the possibility of including grade 3 EECs with other Type II EECs. However, it is uncertain if these molecular signatures are associated with or can differentially predict response to chemotherapy in endometrioid cancers and Type II cancers. Additional analyses into molecular fingerprints of these malignancies have also helped to characterize pathways that may be involved in low grade, yet aggressive EEC in young women. While the PI3K pathway is a source of frequent mutations in EEC, differential expression of hotspot mutations have been noted in microsatellite stable vs. instable endometrial tumors [16]. As we learn more about the intricacies of the cancer genome, it is apparent that histology and tumor grade may not be the only factors that determine the behavior of these endometrial malignancies.

Limitations of this study include the lack of size equivalence between the tumor grade cohorts (namely, fewer tumors in the grade 1 cohort). In addition, not all tumors underwent chemosensitivity testing to all 7 agents as physicians could choose which drugs to submit, thus introducing a selection bias. Strengths of this study include the prospective collection of data that was routinely and comprehensively monitored, as well as the uniform preparation and performance of the chemosensitivity assay. Less than 20% of submitted samples fail the assay due to insufficient cell growth in culture or contamination. We were unable to assess the association between in vitro response results and clinical RR and survival outcomes due to limited clinical data.

## Conclusion

Based on our results, there does not appear to be an association between tumor grade and in vitro chemoresponse assay results. Specifically low grade EECs are not more likely to have resistant assay results compared to higher grade EECs. In addition, 50% of grade 1 EECs demonstrated in vitro sensitivity to at least one cytotoxic therapy, suggesting that chemotherapy may be useful in advanced low grade EECs. However, further clinical correlation is needed to assess assay sensitivity/resistance to in vivo response and clinical outcomes to determine if chemoresponse assays may be useful to direct therapy in women with endometrioid endometrial cancer.

## Abbreviations

AUC: Area under the dose response curve
C/T: Carboplatin/paclitaxel
EC: Endometrial cancer
EEC: Endometrioid endometrial cancer
EOC: Epithelial ovarian cancer
I: Intermediate
R: Resistant
RR: Response Rate
S: Sensitive
TAP: Cisplatin/doxorubicin/paclitaxel
